# Echocardiographic Evaluation of Patent Ductus Arteriosus in Preterm Infants

**DOI:** 10.3389/fped.2017.00147

**Published:** 2017-06-21

**Authors:** Romaine Arlettaz

**Affiliations:** ^1^Department of Neonatology, University Hospital Zurich, Zurich, Switzerland

**Keywords:** preterm infant, patent ductus arteriosus, functional echocardiography, extremely low birth weight infant, ductus arteriosus

## Abstract

Patent ductus arteriosus (PDA) is part of the typical morbidity profile of the preterm infant, with a high incidence of 80–90% in extremely low birth weight infants born before 26 weeks of gestation. Whereas spontaneous closure of the ductus arteriosus (DA) is likely in term infants, it is less so in preterm ones. PDA is associated with increased mortality and various comorbidities including cardiac failure, need for respiratory support, bronchopulmonary dysplasia, pulmonary or intracranial hemorrhage, and necrotizing enterocolitis; however, there is no proven causality between these morbidities and the presence of DA. Thus, the indication to close PDA remains highly controversial. This paper focuses on echocardiographic evaluation of PDA in the preterm infant and particularly on the echocardiographic signs of hemodynamic significance.

## Introduction

Preterm infants account for 6–12% of the newborn population and represent the main group of infants treated in neonatal intensive care units. Among the typical complications observed in preterm infants, patent ductus arteriosus (PDA) is frequent. Its incidence is inversely proportional to the preterm infant’s gestational age, with a rate of about 20% in premature infants born at 32 weeks of gestation compared to an estimated 80–90% in extremely low birth weight infants with a gestational age of less than 26 weeks (1, 2). The likelihood of spontaneous closure of the ductus arteriosus (DA) in full-term infants without congenital heart disease is very high; in the preterm infant, closure rates are poorer (1–3). However, recent studies have shown that PDA will have spontaneously closed by the end of the first week of life in about 73% of preterm infants with a gestational age of 28 weeks (3).

According to the international literature, PDA is associated with not only increased mortality but also various neonatal comorbidities (1, 2, 4, 5). These include cardiac failure, need for respiratory support, need for supplemental oxygen, bronchopulmonary dysplasia, pulmonary hemorrhage, intraventricular hemorrhage, abnormal cerebral perfusion, and necrotizing enterocolitis. Against this background, it became common practice in 1990s to follow a strategy of aggressive medicamentous or surgical closure of PDA in very preterm infants. Nowadays, we know that there is no real proven causality between mortality, neonatal morbidities, and PDA (2, 5). The indications to close PDA have, therefore, decreased dramatically, and the procedure remains controversial. The current approach is to limit treatment to infants most likely to benefit from intervention, i.e., extremely low birth weight infants, particularly those on respiratory support and at risk of rapidly developing a hemodynamically significant PDA (hsPDA) (5).

As clinical signs of PDA are not sensitive or specific enough, all extremely preterm infants at risk of developing PDA should undergo echocardiography. The main goal of the present review is to define and develop the different steps in echocardiographic evaluation of PDA in the preterm infant.

## Echocardiographic Evaluation of PDA

### General Considerations

It is important to consider some general guidelines when evaluating PDA. Although DA is the focus of functional echocardiography, the baby should always undergo a complete echocardiogram first. An initial echocardiogram enables (i) structural congenital heart disease to be excluded, (ii) biventricular function to be measured, and (iii) different aspects of transitional circulation to be checked, as for example pulmonary pressures.

When performing functional echocardiography, it is also important to bear in mind the difficulties and challenges associated with scanning a premature infant. Some babies are very small, they can be agitated or unstable, have a high heart rate, and sometimes a poor echo window, particularly if they are ventilated and have severe underlying lung disease. Some simple tricks include the following: place the baby in the best position (slightly turned to the left with a slightly extended neck), scan the baby when it is in a quiet and stable state whenever possible, and allow plenty of time.

### Specific Evaluation of PDA

Echocardiographic evaluation of PDA in the preterm infant will help physicians answer the following questions: Is the DA patent? What is its size? What is the direction of the shunt? Is the PDA hemodynamically significant?

#### Ductal Patency

**Figure 1:**
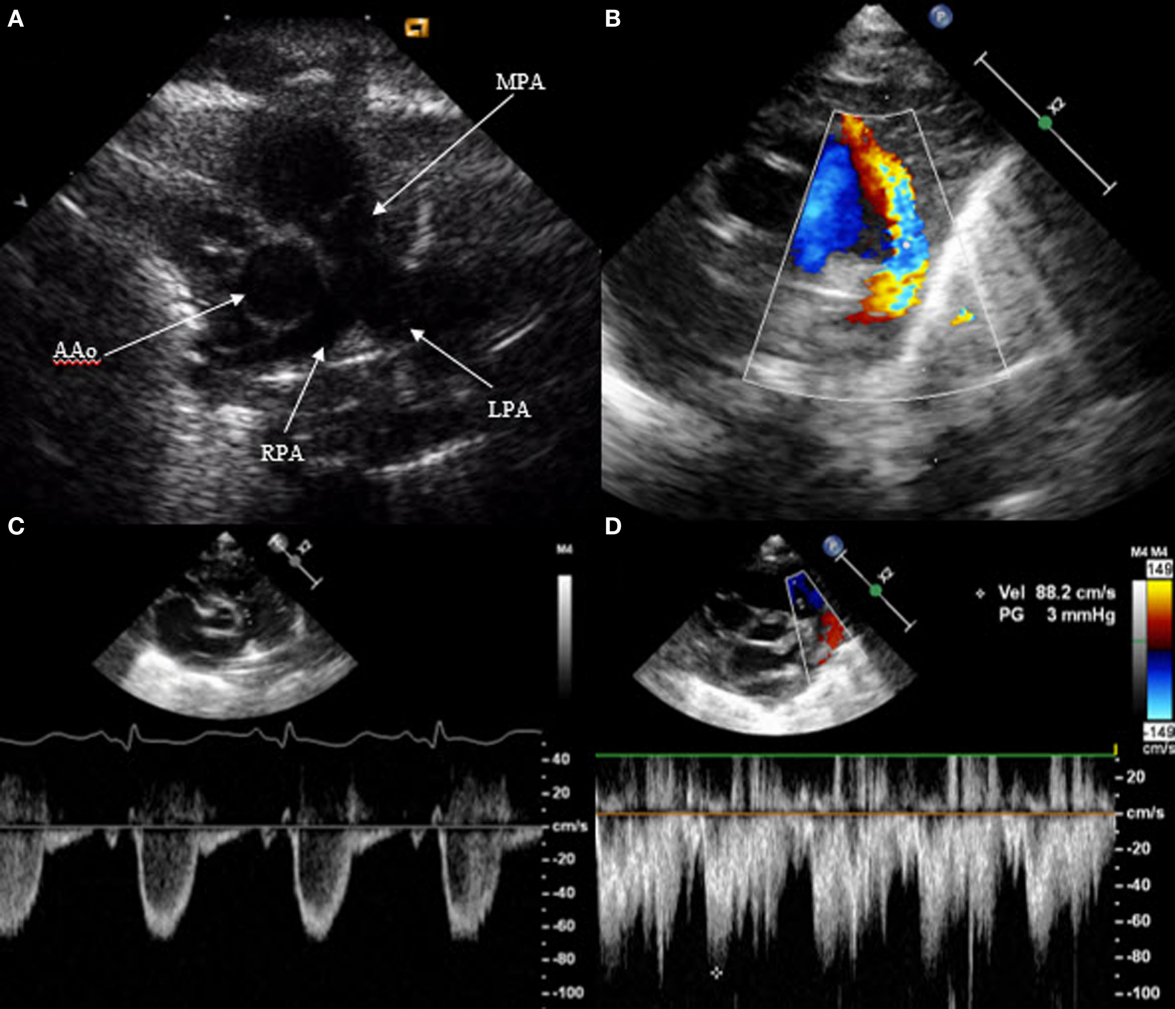
Visualization of patent ductus arteriosus in short axis view (SAX). **(A)** 2D SAX moved anteriorly, **(B)** same picture with color Doppler, **(C)** Doppler in the main pulmonary artery by closed duct, and **(D)** Doppler in the main pulmonary artery by patent duct. MPA, main pulmonary artery; RPA, right pulmonary artery; LPA, left pulmonary artery; AAo, ascending aorta.

**Figure 2:**
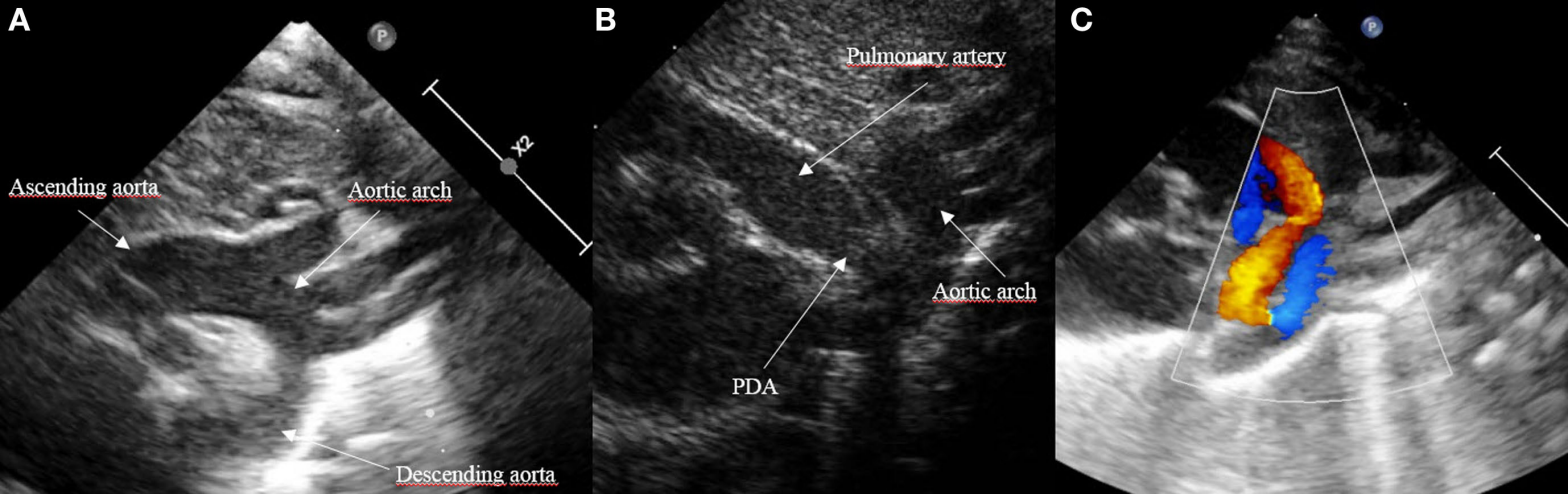
Visualization of patent ductus arteriosus (PDA) in suprasternal view. **(A)** Normal suprasternal view of the aortic arch, **(B)** suprasternal sagittal view with pulmonary artery and descending aorta, and **(C)** color Doppler in a sagittal suprasternal view with PDA in red.

Patent ductus arteriosus can be seen on each of the classic echo views, but the most preferred views are the parasternal short axis view (SAX) and the suprasternal view. With SAX, the DA is visualized at the base of the heart by moving the probe slightly anteriorly toward the pulmonary artery. A small DA can easily be missed on 2D pictures, thus pulsed wave Doppler and color Doppler are helpful (Figure 1). Pulsed wave Doppler in the main pulmonary artery shows a quiet flow when the DA has closed and turbulent systolic and diastolic flow when DA is patent (Figure 1). For the suprasternal view, the probe should be placed at a sagittal level at the midline in order to visualize the pulmonary artery on the left and the descending aorta on the right of the picture. The DA appears between both great arteries (Figure 2).

**Figure 3:**
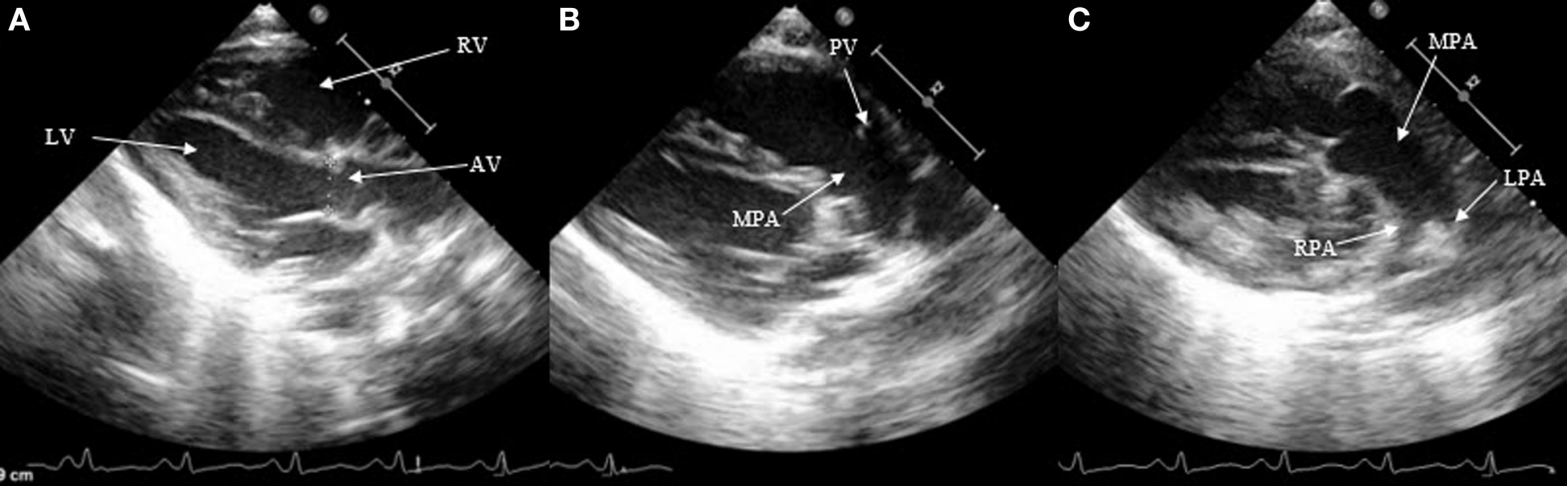
Visualization of patent ductus arteriosus in long axis view (LAX). **(A)** 2D classical LAX, **(B)** LAX slightly tilted anteriorly toward the pulmonary artery, and **(C)** main pulmonary artery with pulmonary branches. RV, right ventricle; LV, left ventricle; AV, aortic valve; PV, pulmonary valve; MPA, main pulmonary artery; RPA, right pulmonary artery; LPA, left pulmonary artery.

Besides the SAX and suprasternal views, it is also possible to see the DA in the parasternal long axis view (LAX) by moving the probe from the classic LAX anteriorly toward the pulmonary artery (Figure 3). The subcostal LAX also allows the detection of the DA. The only window which is not appropriate for detecting DA is the apical four chamber view.

#### Ductal Size

**Figure 4:**
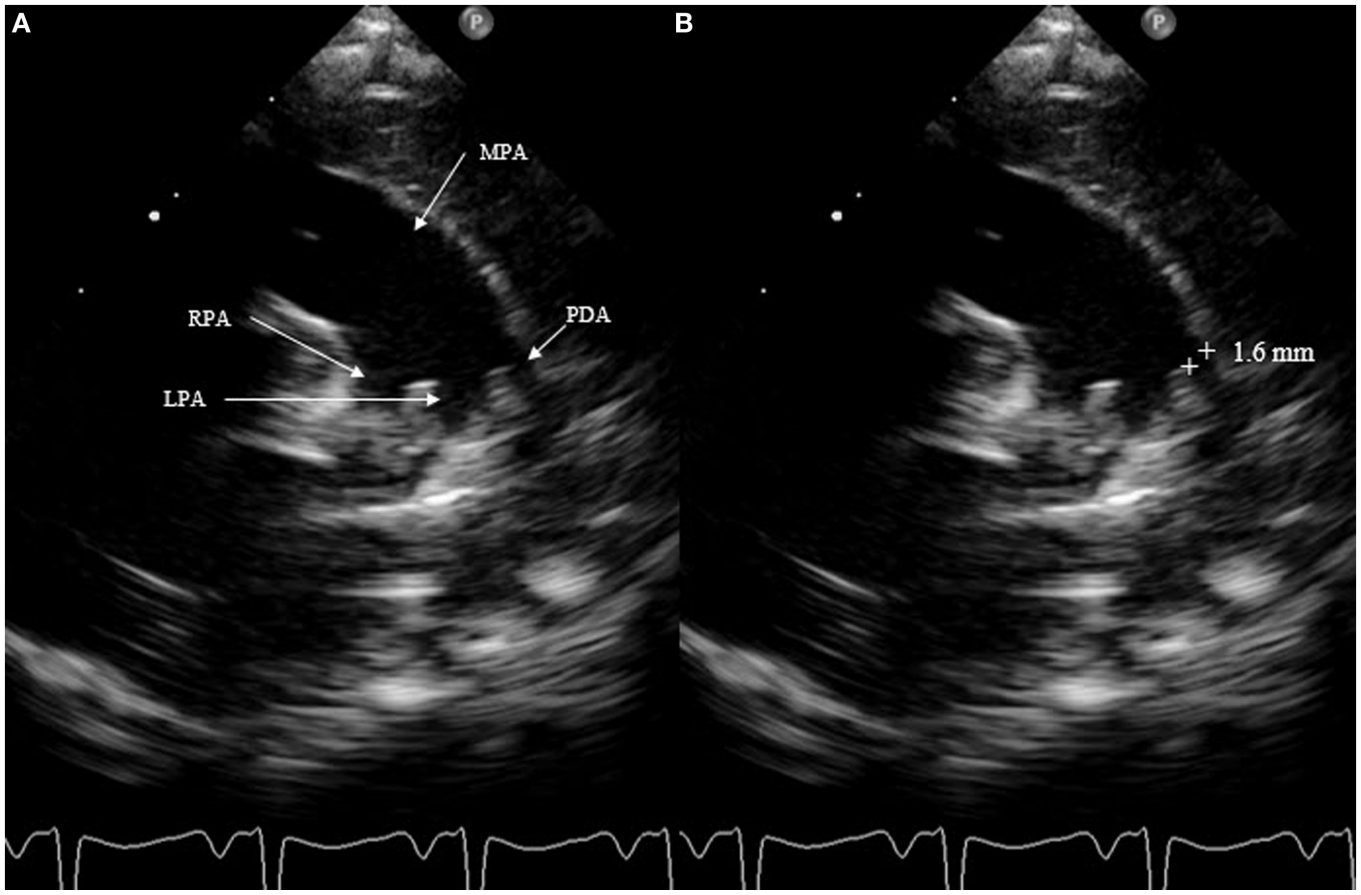
Measurement of patent ductus arteriosus (PDA) size on suprasternal view. MPA, main pulmonary artery; RPA, right pulmonary artery; LPA, left pulmonary artery.

The most accurate view for measuring the size of the DA is the high left parasternal short axis window, also called “ductal view.” Concentrating on the main pulmonary artery, the origin of the right pulmonary artery, which is not always visible, and of the left pulmonary artery can be visualized; the DA is positioned to the left of it. The DA should be measured at its narrowest point, before its entry into the main pulmonary artery (Figure 4).

Measurement of the ductal size is not very accurate. It is not recommended to use the color Doppler, which can exaggerate the size. Measurements must always be performed using a consistent technique. A PDA is considered small at <1.5 mm, moderate when it ranges between 1.5 and 3 mm, and large if the dimension exceeds 3 mm. SAX should not be chosen to measure ductal size because it is not always possible to differentiate the left pulmonary artery from the DA.

#### Direction of the Shunt across the PDA

**Figure 5:**
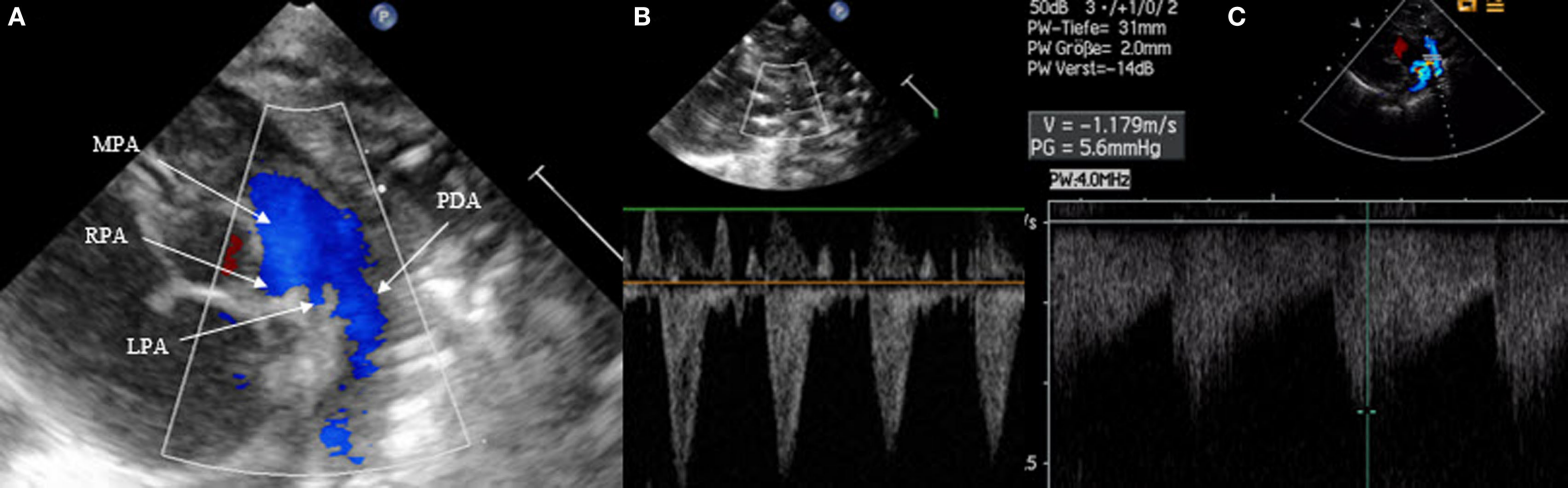
Right-to-left shunt across the patent ductus arteriosus (PDA). **(A)** Short axis view; color Doppler flow pattern is blue during systole and diastole, **(B)** flow across the PDA is below the baseline during systole, and **(C)** flow across the PDA is below the baseline during systole and diastole in the case of “pure right-to-left shunt.” MPA, main pulmonary artery; RPA, right pulmonary artery; LPA, left pulmonary artery.

**Figure 6:**
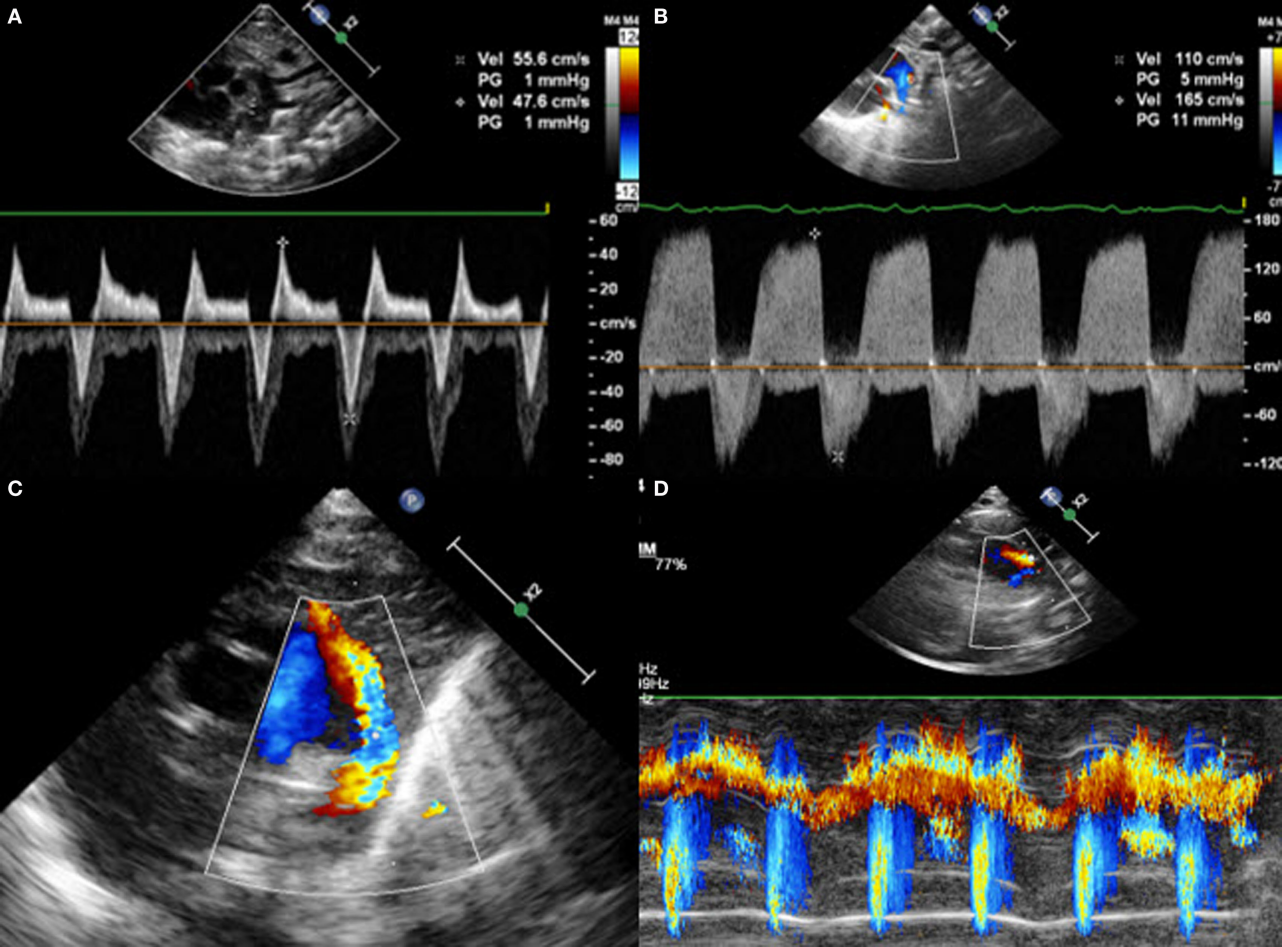
Suprasternal view with **(A)** bidirectional shunt, **(B)** bidirectional shunt predominantly left-to-right, **(C)** left-to-right shunt on color Doppler, and **(D)** left-to-right shunt on color Doppler M-Mode.

The direction of the shunt across the PDA can be right to left, bidirectional, or left to right. In order to document it, color Doppler is required. A right-to-left shunt across the PDA is more difficult to see: color Doppler will show a flow going from the pulmonary artery toward the descending aorta. Thus, both great arteries and the DA will appear blue on color Doppler because the blood moves away from the transducer. Placing pulsed wave Doppler over the DA shows a Doppler wave below the baseline, usually during systole. Less often, a continuous flow during systole and diastole can be observed, representing a pure right-to-left shunt (Figure 5). When the pulmonary vascular resistance falls, the shunt will be bidirectional with a flow above the baseline during the systole and below the baseline during the diastole (Figure 6). Velocities are usually low and suggest equal pressure in the pulmonary artery and the descending aorta. Color Doppler shows alternating red and blue. Once the pulmonary pressure drops further, the shunt will be left to right and appears red on color Doppler. On pulsed or continuous wave Doppler, maximal velocities during systole and diastole can be measured, see below. It is also possible to demonstrate shunt direction on color Doppler using M-Mode (Figure 6).

#### Is the PDA Hemodynamically Significant?

In a physiological state, the pulmonary to systemic flow ratio (Qp:Qs) is 1. The right ventricular output reflects the systemic blood flow whereas the left ventricular output reflects the pulmonary blood flow. The shunt across the patent foramen ovale influences the right ventricular output, and the shunt across the PDA influences the left ventricular output. Thus, ventricular output measurements in a newborn infant with open DA and foramen ovale are not very accurate.

In the case of hsPDA, the pulmonary blood flow increases due to the amount of blood running back from the descending aorta into the lung circulation through PDA; this pattern is known as steal phenomenon. At the same time, the systemic blood flow decreases for the same reason. Qp:Qs ratio will increase.

There is no clear clinical definition of hsPDA. Sosenko et al. (6) mentioned hsPDA in the presence of clinical signs of PDA associated either with pulmonary hemorrhage, or with cardiomegaly and signs of pulmonary edema, as well as respiratory failure and/or arterial hypotension requiring vasopressor treatment. McNamara and Sehgal (7) proposed a staging system with a comparison between clinical and echocardiographic criteria of hsPDA.

The main echocardiographic criteria mentioned in the literature include an enlargement of the left atrium with a left atrium to aortic valve (LA:Ao) ratio of ≥1.5, and absent or retrograde diastolic flow in the descending aorta, absent or retrograde diastolic flow in the mesenteric superior artery and/or in the anterior cerebral artery, a moderate to large PDA diameter of ≥ 1.5 mm at the narrowest point, and an unrestrictive pulsatile transductal flow (2, 5, 6, 8–12).

#### How Can We Measure These Parameters?

**Figure 7:**
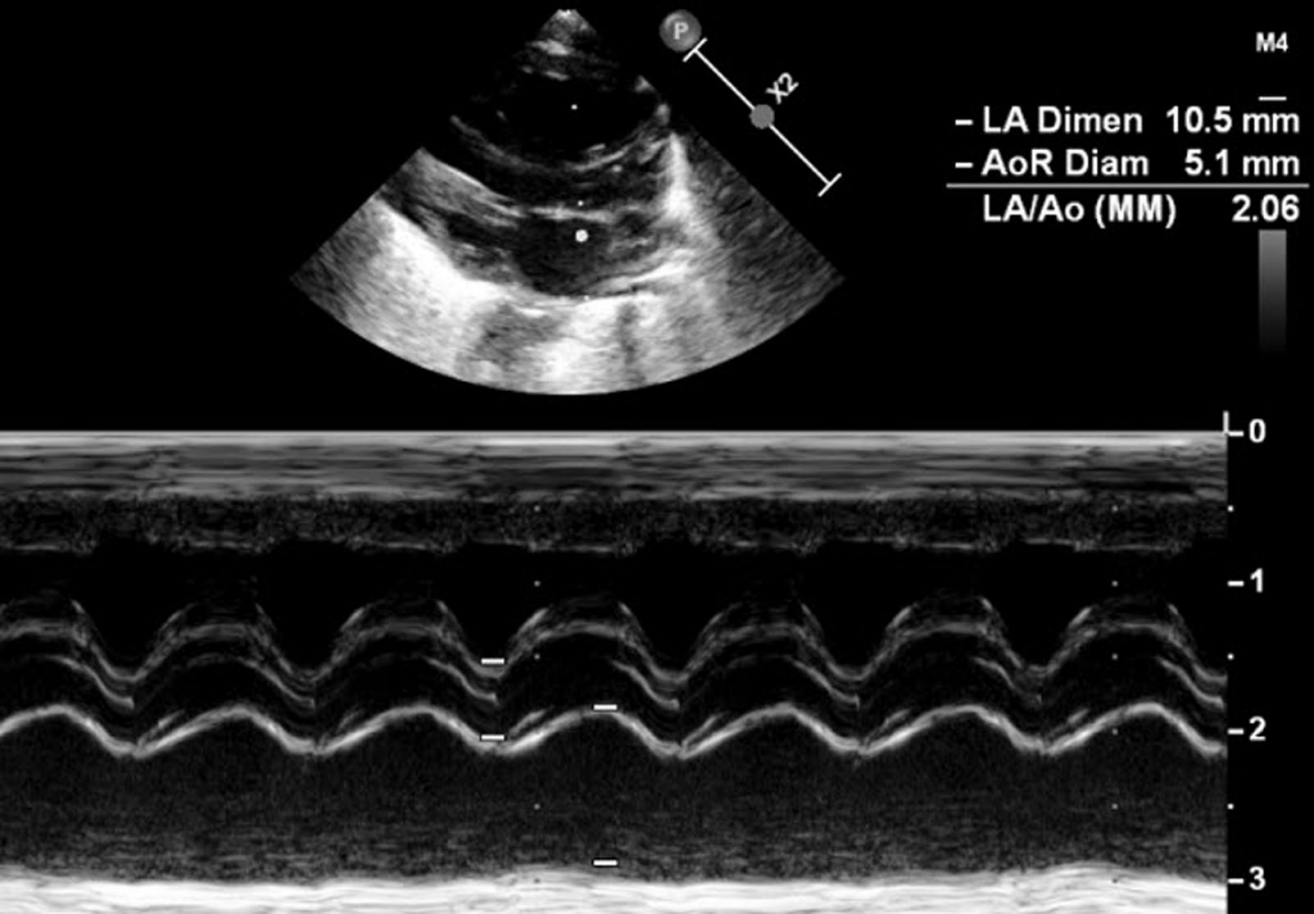
Measurement of LA:Ao ratio in parasternal long axis view in M-Mode.

LA:Ao ratio is measured on parasternal LAX using M-Mode (Figure 7). A cut is made in the left atrium at the level of the aortic valve, with the transducer placed perpendicular to it. The aortic valve is measured just before its opening, at the end of the diastole, whereas the left atrium is measured at its maximal volume during the systole. The LA:Ao ratio should not exceed 1.5. This measurement is easy to perform but not very accurate for two reasons: first, the left atrium is not always easy to delimitate, particularly because of the entrance of the pulmonary veins into it. Second, the atrial shunt influences LA:Ao ratio as a large foramen ovale or atrial septal defect will allow the engorged left atrium to empty into the right one.

**Figure 8:**
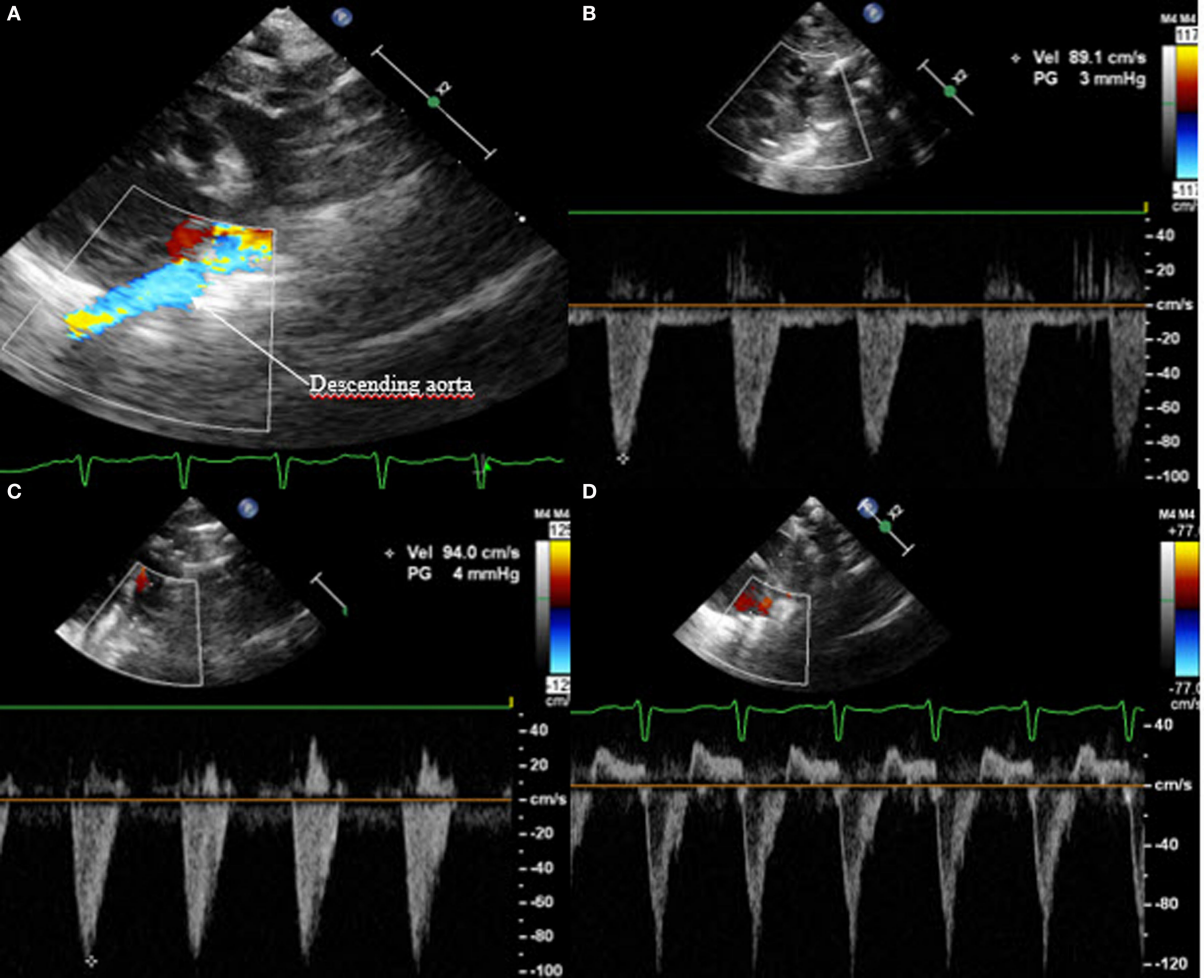
Suprasternal view: **(A)** color Doppler of the postductal descending aorta with **(B)** normal anterograde flow, **(C)** absent end diastolic flow, and **(D)** pandiastolic retrograde flow.

A further sign of hsPDA is the absent or retrograde diastolic flow in the descending aorta. In order to visualize this, the transducer has to be placed in the postductal descending aorta; the pulsed wave Doppler is used. In a physiological situation, the Doppler wave is anterograde during the systole and the diastole, with the wave seen under the baseline. When the steal phenomenon appears, the end diastolic flow will be absent, and if the Qp:Qs ratio further increases, the flow will be retrograde during the whole diastole, appearing above the baseline (Figure 8). This measurement is one of the most specific in evaluating the hemodynamic significance of PDA.

**Figure 9:**
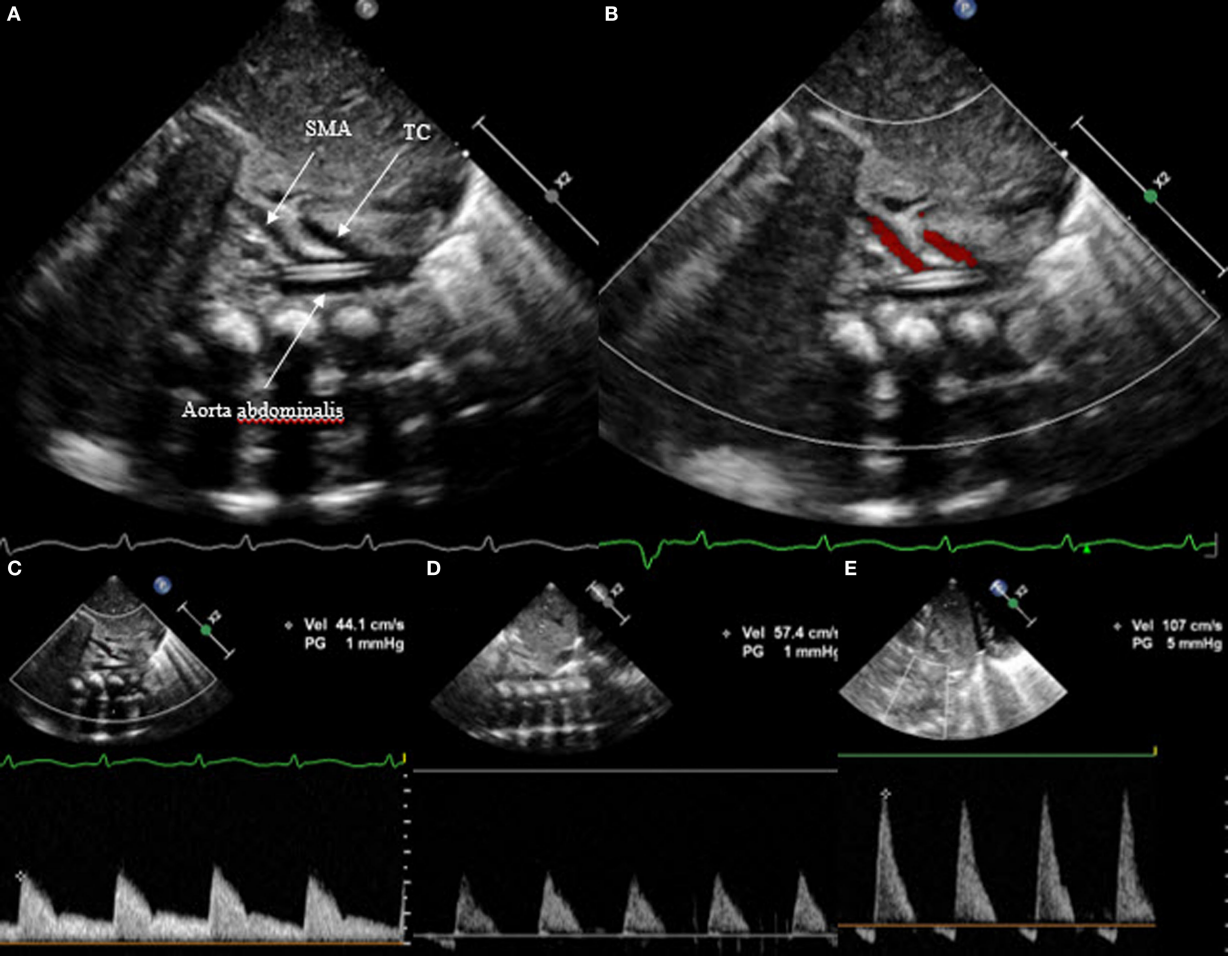
Subcostal view of the descending aorta abdominalis with visualization of the truncus coeliacus and superior mesenteric artery, **(A)** on 2D, **(B)** on color Doppler, **(C)** with anterograde flow, **(D)** with absent end diastolic flow, and **(E)** with retrograde flow. TC, truncus coeliacus; SMA, superior mesenteric artery.

In a similar way, the diastolic flow is measured in the abdominal descending aorta and in the mesenteric superior artery. Again, the diastolic flow should be anterograde and is seen as a Doppler curve above the baseline. In the case of hsPDA, it can be absent or even appear retrograde (Figure 9). In this case, the perfusion of the small intestine is assumed to be compromised.

**Figure 10:**
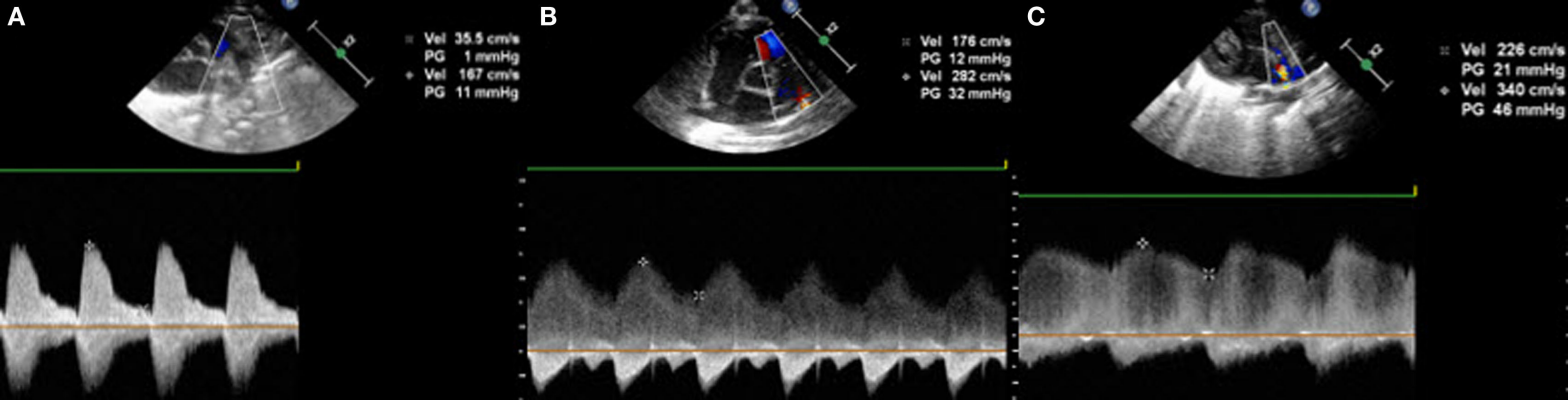
Transductal flow on Doppler in short axis view or suprasternal view: **(A,B)** unrestrictive pulsatile flow and **(C)** restrictive continuous flow.

Last but not least, the Doppler flow pattern across the PDA is measured. This will show one of three patterns (Figure 10). Non-hemodynamically significant PDA has a high flow velocity during systole and diastole with a maximal velocity above 2 m/s at the end of the diastole; this is called “restrictive continuous transductal flow.” If the PDA is moderately hemodynamically significant, the Doppler flow pattern has a maximal diastolic velocity of less than 2 m/s and is called “unrestrictive pulsatile transductal flow.” In a large hsPDA, the flow is also an “unrestrictive pulsatile transductal flow” but the maximal end-diastolic velocity is below 1 m/s. Next to the absolute values, it is also possible to measure the ratio between the systolic and diastolic velocities. If the peak diastolic velocity is more than 50% of the peak systolic velocity, the flow pattern is restrictive. If this ratio is less than 50%, the flow pattern is pulsatile suggesting hemodynamically significant PDA.

#### Summary of Echocardiographic Parameters of PDA

The most specific echocardiographic parameters of PDA can be divided into three groups: (i) the ductal characteristics, (ii) overcirculation of the lungs, and (iii) systemic hypoperfusion. The latter two are observed in the case of hsPDA.

##### The Characteristics of PDA

The characteristics of PDA are best evaluated by measuring the ductal size and the velocities across the DA, both in a suprasternal sagittal view. The ductal diameter measured on the first day of life has a high sensitivity and specificity in predicting the development of hsPDA (2). Further authors also describe the association between ductal size and the need for further medicamentous or surgical closure (13). Measuring flow velocities from the suprasternal view gives information about the direction of the shunt and allows pulmonary pressures to be assessed. Furthermore, quantification of the systolic and diastolic flow velocities across the PDA and particularly the peak systolic to end diastolic flow velocity ratio is a good predictor of ductal patency in preterm infants born before 32 weeks of gestation (14).

##### Pulmonary Hyperperfusion

Pulmonary hyperperfusion is best documented with a LA:Ao ratio of ≥1.5, an increased flow across the mean pulmonary artery, an elevated diastolic flow in the left pulmonary artery, and a mitral E/A ratio of >1.5 suggesting severe left heart pressure loading (2, 7). The most popular echocardiographic parameter is the LA:Ao ratio which is easy to measure. However, this parameter is influenced by the shunt across the patent foramen ovale.

##### Systemic Hypoperfusion

Systemic hypoperfusion is best evaluated with the presence of a retrograde flow in the descending aorta as well as in the anterior cerebral and the superior mesenteric arteries.

These parameters help to grade PDA as non-symptomatic, or mildly, moderately, or severely hemodynamically significant. The literature fails to agree on the best parameters for assessing ductal significance, but Condo et al. suggested ductal size and flow patterns to be the most sensitive (9). However, these parameters have to be related to the clinical symptoms presented by the baby as well as its postnatal age. The hemodynamic transition, characterized by an elevated pulmonary vascular resistance, will be reflected on echocardiography by a predominantly right-to-left shunt in the first minutes of life, followed by a bidirectional shunt and later a left-to-right shunt with low ductal flow velocities (15). With the physiological lowering and normalization of the pulmonary pressures, an increase in the systolic and diastolic velocities is observed. Also, the ductal size measured in the first 2 days of life is a good predictor of development of symptomatic DA, which is not the case later in life. Thus, the approach used in assessing hsPDA varies according to the timing of echo.

## Discussion

Echocardiographic evaluation of PDA in very preterm infants is one of the everyday activities in a neonatal intensive care unit and is usually performed by not only pediatric cardiologists but also by neonatologists or pediatric intensivists trained in functional echocardiography. It is not easy to perform echocardiograms in extremely preterm infants, and the fact that there is intra- as well as interobserver variability is certainly not optimal. Thus, it is important to follow some basic rules. First, echocardiogram can focus on PDA only after a previous complete echo has excluded structural congenital heart disease and measured ventricular function. Second, scanning a preterm infant can be challenging, and the evaluation of PDA is time consuming, so it is also important to keep in mind the general considerations described in this review.

If the echocardiogram of a preterm infant with PDA has been performed by a neonatologist or intensivist, the echocardiographic findings should be confirmed by a pediatric cardiologist and a joint decision should be taken regarding treatment of PDA and follow-up of the baby.

The debate on whether or not to treat PDA is beyond the scope of the present review. However, many authors suggest treating only babies presenting with hsPDA and showing clinical signs of respiratory failure. Indeed, a baby doing very well and not requiring ventilator support or supplemental oxygen will usually not benefit from ductal closure. In this sense, echocardiographic evaluation of PDA should be accompanied by the definition of its clinical significance. One possible approach is the staging system published 10 years ago by McNamara and Sehgal (7), setting out a detailed correlation between the clinical and echocardiographic signs. Neonatologists may find this helpful in deciding if and when to start medicamentous treatment in order to close PDA.

## Author Contributions

RA is the only author and has fully contributed to the manuscript.

## Conflict of Interest Statement

The author declares that the research was conducted in the absence of any commercial or financial relationships that could be construed as a potential conflict of interest.

## Abbreviations

DA, ductus arteriosus; hsPDA, hemodynamically significant patent ductus arteriosus; LA:Ao, left atrium to aortic valve; LAX, long axis view; PDA, patent ductus arteriosus; SAX, short axis view.
